# Synthetic faces generated with the facial action coding system or deep neural networks improve speech-in-noise perception, but not as much as real faces

**DOI:** 10.3389/fnins.2024.1379988

**Published:** 2024-05-09

**Authors:** Yingjia Yu, Anastasia Lado, Yue Zhang, John F. Magnotti, Michael S. Beauchamp

**Affiliations:** ^1^Department of Neurosurgery, Perelman School of Medicine, University of Pennsylvania, Philadelphia, PA, United States; ^2^Department of Neurosurgery, Baylor College of Medicine, Houston, TX, United States

**Keywords:** audiovisual, multisensory, speech, face, speech-in-noise (SIN), deep neural

## Abstract

The prevalence of synthetic talking faces in both commercial and academic environments is increasing as the technology to generate them grows more powerful and available. While it has long been known that seeing the face of the talker improves human perception of speech-in-noise, recent studies have shown that synthetic talking faces generated by deep neural networks (DNNs) are also able to improve human perception of speech-in-noise. However, in previous studies the benefit provided by DNN synthetic faces was only about half that of real human talkers. We sought to determine whether synthetic talking faces generated by an alternative method would provide a greater perceptual benefit. The facial action coding system (FACS) is a comprehensive system for measuring visually discernible facial movements. Because the action units that comprise FACS are linked to specific muscle groups, synthetic talking faces generated by FACS might have greater verisimilitude than DNN synthetic faces which do not reference an explicit model of the facial musculature. We tested the ability of human observers to identity speech-in-noise accompanied by a blank screen; the real face of the talker; and synthetic talking faces generated either by DNN or FACS. We replicated previous findings of a large benefit for seeing the face of a real talker for speech-in-noise perception and a smaller benefit for DNN synthetic faces. FACS faces also improved perception, but only to the same degree as DNN faces. Analysis at the phoneme level showed that the performance of DNN and FACS faces was particularly poor for phonemes that involve interactions between the teeth and lips, such as /f/, /v/, and /th/. Inspection of single video frames revealed that the characteristic visual features for these phonemes were weak or absent in synthetic faces. Modeling the real vs. synthetic difference showed that increasing the realism of a few phonemes could substantially increase the overall perceptual benefit of synthetic faces.

## Introduction

Recent advances in computer graphics have made it much easier to create realistic, synthetic talking faces, spurring adoption in commercial and academic communities. For companies, agents created by pairing a synthetic talking face with the output of large language models provide an always-available simulacrum of a real human representative (Perry et al., 2023). In academia, the use of synthetic talking faces in studies of speech perception provides more precise control over the visual features of experimental stimuli than is possible with videos of real human talkers (Thézé et al., 2020a).

Of particular interest is the long-standing observation that humans understand speech-in-noise much better when it is paired with a video of the talker’s face (Sumby and Pollack, 1954). The ability to rapidly generate a synthetic face saying arbitrary words suggests the possibility of an “audiovisual hearing aid” that displays a synthetic talking face to improve comprehension. This possibility received support from two recent studies that used deep neural networks (DNNs) to generate realistic, synthetic talking faces (Shan et al., 2022; Varano et al., 2022). Both studies found that viewing synthetic faces significantly improved speech-in-noise perception, but the benefit was only about half as much as viewing a real human talker.

The substantial disadvantage of synthetic faces raises the question of whether alternative techniques for generating synthetic faces might provide a greater perceptual benefit. DNNs associate given speech sounds with visual features in their training dataset, but do not contain any explicit models of the facial musculature. In contrast, the facial action coding system (FACS) uses 46 basic action units to represent all possible movements of the facial musculature that are visually discernable (Ekman and Friesen, 1976, 1978; Parke and Waters, 2008). Unlike DNNs, the FACS scheme is built on an understanding of the physical relationship between speech and facial anatomy, potentially resulting in more accurate representations of speech movements. To test this idea, we undertook a behavioral study to compare the perception of speech-in-noise on its own; speech-in-noise with real faces (to serve as a benchmark); and speech-in-noise presented with two types of synthetic faces. The first synthetic face type was generated by a deep neural network, as in the studies of (Shan et al., 2022; Varano et al., 2022). The second synthetic face type was generated using FACS, as implemented in the commercial software package JALI (Edwards et al., 2016; Zhou et al., 2018). For comparison with previous studies, we performed a word-level analysis in which each response was scored as correct or incorrect. To facilitate more fine-grained comparisons between the different face types, we also analyzed data using the phonemic content of each stimulus word.

## Methods

### Participant recruitment and testing

All experiments were approved by the Institutional Review Board of the University of Pennsylvania, Philadelphia, PA. Participants were recruited and tested using Amazon Mechanical Turk,^1^ an online platform that provides access to an on-demand workforce. Only “master workers” were recruited, classified as such by Amazon based on their high performance and location in the United States. Sixty-two participants completed the main experiment (median time to complete: 12 min) and received $5 reimbursement. The participants answered the questions “Do you have a hearing impairment that would make it difficult to understand words embedded in background noise?” and “Do you have an uncorrected vision impairment that would make it difficult to watch a video of a person talking?” One participant was excluded because of a reported hearing impairment, leaving 61 participants whose data is reported here. There were 25 females and 36 males, mean age 46 years, range 30–72.

### Overview

Workers were asked to enroll in the experiment only if they were using a desktop or laptop PC or a tablet (but not a phone) and information about the user’s system was collected to verify compliance. At the beginning of the experiment, participants viewed an instructional video (recorded by author MSB) that explained the task and presented examples of the different experimental stimuli. The instructional video was accompanied by text instructions stating “Please adjust your window size and audio volume so that you can see and hear everything clearly.”

**Figure 1:**
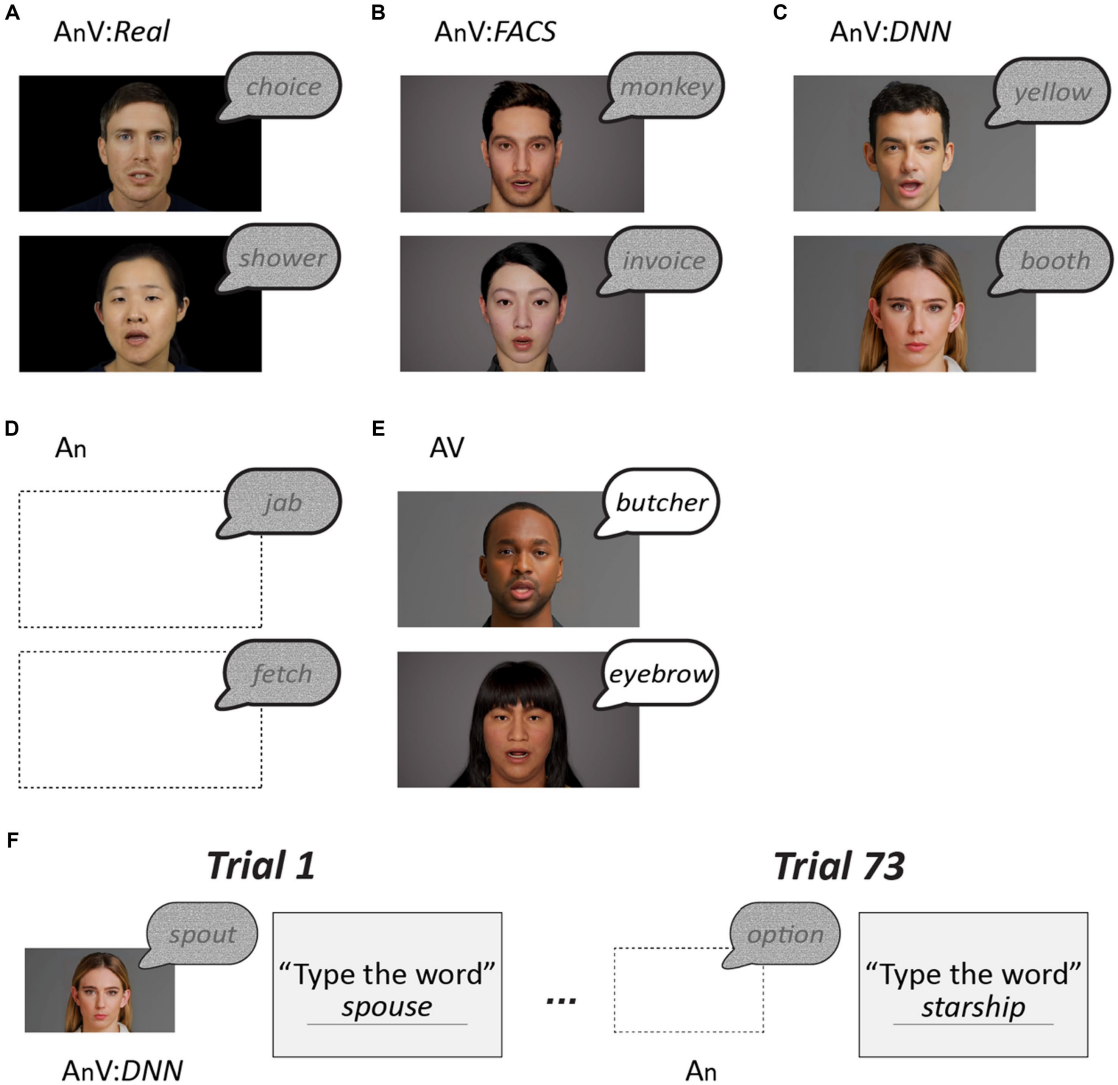
**(A)** The main stimulus set consisted of 64 single words with auditory noise added, 32 recorded by a male talker and 32 recorded by a female talker. The noisy words were presented in four formats. The first format consisted of the noisy auditory recordings paired with a video of the actual talker (AnV:*Real*). **(B)** The second format consisted of the recordings paired with gender-matched synthetic face movies generated by a facial action coding system model (AnV:*FACS*). **(C)** The third format consisted of the recordings paired with synthetic face movies generated by a deep neural network (AnV:*DNN*). The gender of the synthetic face matched the gender of the voice. **(D)** The fourth format consisted of the recordings presented with a blank screen (An). Note that for **(A)**–**(D)**, the auditory component of the stimulus was identical, only the visual component differed. **(E)** In catch trials, an additional stimulus set was presented consisting of recordings of 9 audiovisual words without added noise (AV) paired with gender-matched real, FACS, or DNN faces (three words each). The words, faces and voices were different than in the main stimulus set to prevent any interference. **(F)** Each participant was presented with 73 words (64 noisy and 9 clear) in random order. Within participants, each noisy word from the main stimulus set was presented only once, in one of the four formats. Counterbalancing was used to present every noisy word in each of the four formats shown in **(A)**–**(D)**. For instance, for participant 1, the word *spout* was presented in AnV:*DNN* format, while for participant 2, *spout* was presented in AnV:*Real* format, etc. Following the presentation of each word, participants typed the word in a text box.

Following completion of the instructional video, participants identified 73 words presented in five different formats (Figure 1). Sixty-four of the words contained added auditory noise to make identifying them more difficult and increase the importance of visual speech. There were four formats of noisy words: auditory-only (An); with a talking face (audiovisual; AnV) that was either the real face of the talker (AnV:*Real*); a synthetic face created using the facial action coding system (AnV:*FACS*); or a synthetic face created using a deep neural network (AnV:*DNN*). To prevent perceptual learning, each word was only presented once to each participant, 16 words in each of the four formats. Within participants, the order of words and face formats was randomized, and across participants, the format of each word was cycled to ensure that every word was presented in every format. To assess participant compliance, the remaining nine words presented were clear audiovisual words (AV:*catch_trials*). The catch trials sampled all face types (3 Real, 3 FACS, and 3 DNN) and the talkers and words differed from those presented in the noisy trials to prevent learning. Accuracy for catch trials was very high (mean of 98%) demonstrating attention and task engagement. All data was analyzed in R, primarily using mixed effects models. See Supplementary Material for all data and an R markdown document that contains all analysis code and results.

### Subject responses and scoring: word-level

Following presentation of a word, participants were instructed to “type the word” into a text box; the next trial did not begin until a response was entered. If a participant’s response matched the stimulus word, the trial was scored as “correct,” otherwise the trial was scored as “incorrect.” For example, the stimulus word *wormhole* and the response *wormhole* was correct, while the stimulus word *booth* and the response *boot* was incorrect. Misspellings were not considered incorrect (e.g., stimulus *echos* and response *echoes* was correct) nor were homophones (e.g., stimulus *wore* and response *war* was correct). The analysis was performed separately for each condition (An, AnV:*Real*, AnV:*DNN*, AnV:*FACS*, AV:*catch_trials*). Mean accuracy for each condition was calculated per participant, and then averaged across participants. See Supplementary Material for a complete list of the words, response and scores.

### Phonemic analysis

In addition to the binary word-level accuracy measure, a continuous accuracy measure was calculated for each trial based on the overlap in the phonemes in the stimulus word and the response. Phoneme composition was determined using the Carnegie Mellon University (CMU) pronouncing dictionary.^2^ Lexical stress markers were removed from vowels. For responses that contained multiple words, the phonemes for all response words were extracted from the CMU dictionary and entered into the calculation. Repetitions of the same phoneme were also entered into the calculation. The accuracy measure was the Jaccard index: the number of phonemes in common between the stimulus and response divided by the total number of phonemes in the stimulus and response. The measure ranged from 0 (no phonemes in common between stimulus and response) to 1 (identical phonemes in stimulus and response) and was calculated as


$$\begin{array}{c} J\left(\mathrm{stimulus},\mathrm{response}\right)=\frac{\left|\mathrm{stimulus}\cap \mathrm{response}\right|}{\left|\mathrm{stimulus}\cup \mathrm{response}\right|}\\ =\frac{\left|\mathrm{stimulus}\cap \mathrm{response}\right|}{|\mathrm{stimulus}|+|\mathrm{response}|-|\mathrm{stimulus}\cap \mathrm{response}|} \end{array}$$

For example, the stimulus word *polish* contains the phonemes P, AA, L, IH, SH. One participant’s response was *policy*, containing the phonemes P, AA, L, AH, S, IY. The intersection contains three phonemes (P, AA, L) while the union contains 8 phonemes (P, AA, L, IH, SH, AH, S, IY) for a Jaccard index of 3/8 = 38%. In another example, the stimulus word *ethic* contains the phonemes EH, TH, IH, K while the response *essay* contains the phonemes EH, S, EY. The intersection contained 1 phoneme and the union contained 6 phonemes, for a Jaccard index 1/6 = 17%. See Supplementary material for a complete list of Jaccard indices.

The analysis was performed separately for each stimulus condition. For participant-level analysis, the mean accuracy across all trials was calculated for each participant, and then averaged across participants.

For the phoneme-specific analysis, the dependent variable was the number of times a phoneme was successfully identified vs. the total number of times that phoneme was presented across all words, for each participant, for each movie type (Real, DNN, and FACS). Because participants were not shown all possible phonemes for all movie types, we plot the estimated marginal means and standard errors derived from the GLME.

#### Additional stimulus details

The original stimulus material consisted of 32 audiovisual words recorded by a female talker and 32 words recorded by a male talker. Pink noise was added to the auditory track of each recording at a signal-to-noise ratio (SNR) of −12 dB. For the An format, only the noisy auditory recording was played with no visual stimulus. For the AnV:*Real* format, the audio recording was accompanied by the original video recording. For the synthetic faces, gender-matched synthetic faces roughly approximating the appearance of the real talkers were created.

During the online testing procedure, videos were presented using a custom JavaScript routine that ensured that all stimuli were presented with the same dimensions (height: 490 pixels; width: 872 pixels) regardless of the participant’s device.

AnV:*FACS* words were created using JALI software (Edwards et al., 2016).^3^ The text transcript was manually tuned to create more pronounced mouth movements (e.g., AWLTHOH for although; see Supplementary material for a complete list of the phonetic spelling). Following JALI animation, the mouth movements were manually adjusted with MAYA’s graph editor to better match the mouth movements in the real videos. The edited animation sequence was imported into Unreal Engine 5.10 and rendered as 16:9 images at 50 mm focal length and anti-aliasing with 16 spatial sample count. Image sequences were assembled into mp4 format and aligned with the original audio track in Adobe Premiere. The video frame rates was 24 fps.

AnV:*DNN* words were created with the API for D-ID studio.^4^ Two artificial faces included with D-ID studio were used as the base face. Clear audio files from the real speakers were uploaded, and a static driver face was used to minimize head and neck movements. Eye blinking and watermarks in each movie were removed using Adobe Premiere and exported in mp4 format at 24 fps.

### Power analysis

In a pilot study, the audiovisual benefit for real faces and synthetic (FACS) faces was measured in 34 participants using 60 words from the main experiment. Accuracy for real faces was 23% (*SD* = 13%) greater than synthetic faces. Using this effect size (1.68) in a power analysis with G*Power software estimated that only six participants would be required for 90% power to detect a real vs. synthetic difference (*t*-test, difference between two dependent means). However, because we expected a smaller difference between the two synthetic face types (FACS and DNN), a more conservative effect size estimate of 0.5 was substituted. A corrected-alpha level of 0.0167 (0.05/3, to account for three comparisons) resulted in an estimate of 57 participants for 90% power. In anticipation of excluding some participants, five additional participants were recruited, for a total of 62. Only one participant was excluded, resulting in a final sample size of 61.

## Results

### Participant-level analysis: word accuracy

**Figure 2:**
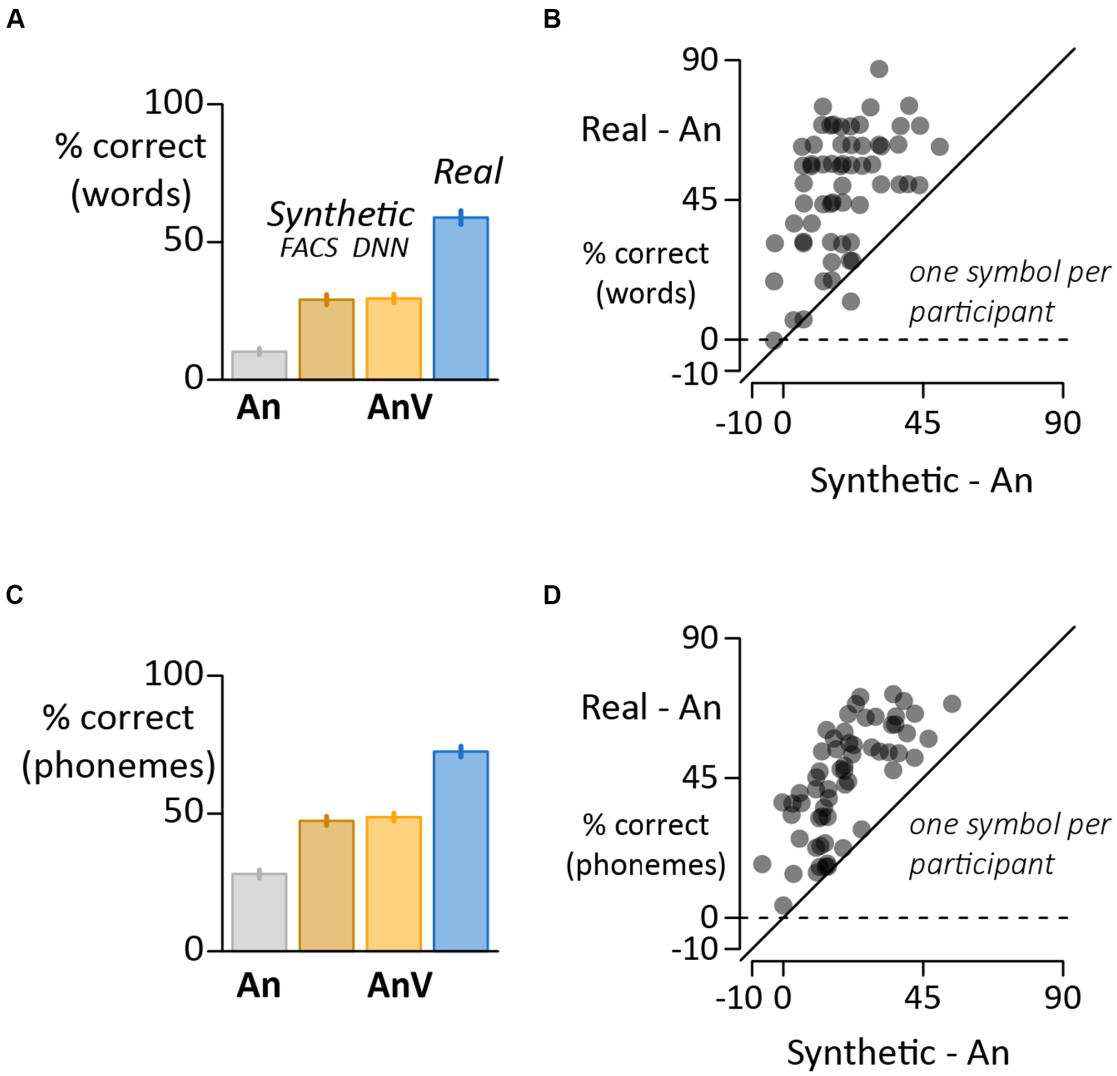
**(A)** For word-level scoring, the response was assessed as correct if it exactly matched the stimulus word and incorrect if it did not. Each bar shows the mean accuracy for each stimulus format (error bars show the standard error of the mean across participants). **(B)** Variability across participants assessed with word-level scoring (one symbol per participant). The *y*-axis shows the perceptual benefit of real faces (real minus auditory-only accuracy). The *x*-axis shows the perceptual benefit of synthetic faces (average of *FACS* and *DNN* accuracies minus auditory-only). Participants above the dashed line show a benefit for real faces compared with auditory-only. Participants above the solid identity line show a greater benefit for real faces than synthetic faces. **(C)** For phoneme-level scoring, the phonemic content of the stimulus and response were compared and the percentage of correct phonemes calculated for each stimulus format. **(D)** Variability across participants assessed with phoneme-level scoring (one symbol per participant).

In the first analysis, responses were scored as correct if they exactly matched the stimulus word and incorrect otherwise. Seeing the face of the talker improved the intelligibility of noisy auditory words. For real faces, accuracy increased from 10% in the auditory-only condition (An) to 59% in the audiovisual condition (AnV:*Real*), averaged across words and participants. There was also an improvement, albeit smaller, for synthetic faces (Figure 2A). From the auditory-only baseline of 10%, accuracy improved to 29% with faces generated by the facial action coding system (AnV: *FACS*). Accuracy was 30% for faces generated by a deep neural network (AnV:*DNN*). While there was a range of accuracies across participants, accuracy was higher for the real face format than the auditory-only format in 60 of 61 participants and higher for real faces than synthetic faces (*Synthetic;* average across *DNN* and *FACS*) in 59 of 61 participants (Figure 2B).

To estimate statistical significance, a mixed-effects model was constructed with a dependent variable of accuracy; fixed effect of word format; and random effects of word, participant and participant batch (complete model specification and output in Supplementary material). There was a main effect of stimulus format (*χ*^2^*_3_* = 768, *p* < 10^−16^) and *post hoc* pair-wise comparisons showed that words accompanied by a visual face (real or synthetic) were perceived more accurately than auditory-only words (all *p* < 10^−16^). The accuracy for real faces was higher than for synthetic faces (*Real* vs. *DNN*; *t* = −17, *p* < 10^−16^; *Real* vs. *FACS*; *t* = −17, *p* < 10^−16^) but accuracy for the two synthetic face formats was equivalent (*DNN* vs. *FACS*; *t* = 0.2, *p* = 0.99).

### Participant-level analysis: phoneme accuracy

In a second analysis, instead of classifying each response as either correct or incorrect, partial credit was given if the response contained phonemes that matched those in the stimulus word. This scoring method generated a phoneme accuracy score for each condition in each participant. The pattern of results were very similar to the word accuracy analysis (Figure 2C Supplementary material). There was a main effect of stimulus format (*χ*^2^*_3_* = 986, *p* < 10^−16^) and *post hoc* pair-wise comparisons showed that words accompanied by a visual face (real or synthetic) were perceived more accurately than auditory-only words (all *p* < 10^−16^). The accuracy for real faces was higher than for synthetic faces (*Real* vs. *DNN*; *t* = −17, *p* < 10^−16^; *Real* vs. *FACS*; *t* = −18, *p* < 10^−16^) but equivalent for the two synthetic face formats (*DNN* vs. *FACS*; *t* = 0.9, *p* = 0.82). Accuracy was higher for real faces than synthetic faces in every participant (Figure 2D).

### Phoneme-level analysis

**Figure 3:**
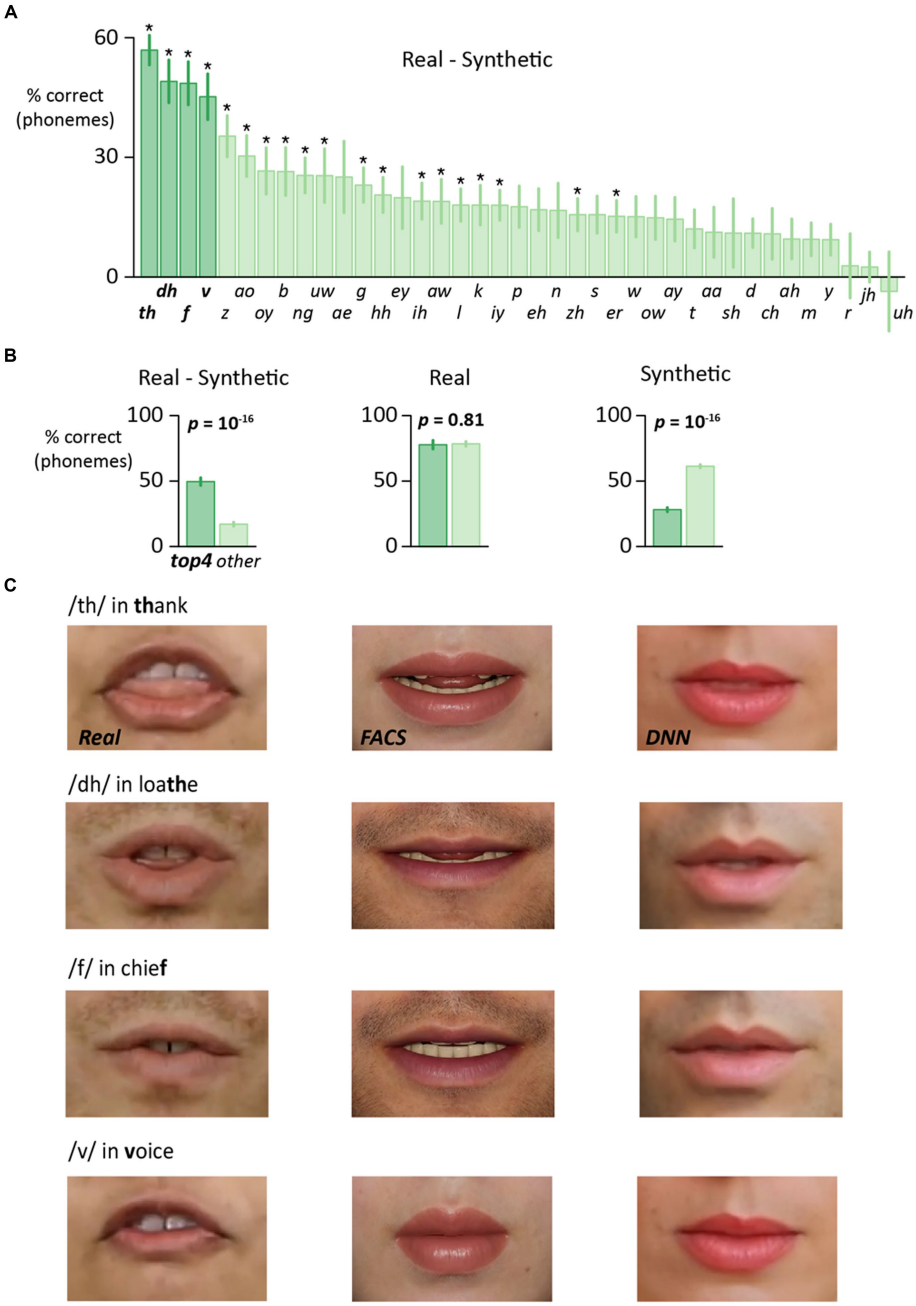
**(A)** For each phoneme, the perceptual accuracy was calculated separately for each stimulus format across all participants. The accuracy for audiovisual synthetic faces (average of *DNN* and *FACS*) was subtracted from the accuracy for audiovisual real faces to generate a single value for each phoneme. For plotting, phonemes were sorted by the difference value. Four phonemes (boldface labels and left four bars colored dark green) showed the largest real-synthetic difference. Phonemes marked with asterisk showed a significantly greater accuracy for real faces after correction for multiple comparisons. **(B)** Four phonemes (*/th/*, */dh/*, */v/*, */f/*) showed the largest real-synthetic difference phonemes (left plot). Average of these four phonemes shown by dark green bar, average of all other phonemes shown by light green bar, error bar shows SEM. This was not due to differences in the real face condition (middle plot) but rather to low accuracy for the top four phonemes in the synthetic face condition (right plot). **(C)** Enlargement of the mouth region for the three different face formats for words containing (*/th/*, */dh/*, */v/*, */f/*). Enlargement for illustration only, participants viewed the entire face, as shown in Figure 1. Top row: video frame 18 from the word *thank*. Second row: video frame 30 from the word *loathe*. Third row: video frame 27 from the word *chief*. Fourth row: Video frame 16 from the word *voice*.

In a third analysis, the accuracy difference between real and synthetic faces was examined separately for each phoneme (Figure 3A). For 38 of 39 phonemes, accuracy was higher for real faces than synthetic faces, and this difference was significant for 19 of 39 phonemes (after Bonferroni-correction for multiple comparisons). For four phonemes (*/th/*, */dh/*, */f/*, */v/*) the accuracy advantage of real faces was especially pronounced (*Real* >> *Synthetic*). This observation could arise because these four phonemes had high accuracy for real faces, low accuracy for synthetic faces, or both. To distinguish these possibilities, we calculated the mean *Real* accuracy for (*/th/*, */dh/*, */f/*, */v/*) compared with other phonemes, and found little difference (78% vs. 78%, *t* = −0.2, *p* = 0.81, paired samples *t*-test). In contrast, the mean *Synthetic* accuracy was significantly lower for (*/th/*, */dh/*, */f/*, */v/*) than for other phonemes (28% vs. 61%, *t* = −25, *p* = 10^−16^). Thus, the greater real-synthetic difference for */th/*, */dh/*, */f/*, */v/* than other phonemes (50% vs. 17%, *t* = 11, *p* = 10^−16^) was attributable to particularly low *Synthetic* accuracy (Figure 3B).

The poor accuracy for synthetic */th/*, */dh/*, */f/*, */v/* suggested that some key visual features might be missing (Figure 3C). For */th/* and */dh/*, the salient visual feature is the tongue sandwiched between the teeth. This feature was clearly visible in the real face videos but was absent from the *DNN* and *FACS* face videos. For */f/* and */v/*, the salient visual feature is the upper teeth pressed onto the lower lip. This feature was obvious in the real face videos but not in the synthetic face videos.

### Modeling the effects of improving the four phonemes

Advances in computer graphics will make it possible to create synthetic faces that more accurately depict */th/*, */dh/*, */f/*, */v/* and thereby increase the synthetic face benefit for words containing these phonemes. To estimate this increase, we constructed a logistic model that predicted the word accuracy based on the presence or absence of every different phoneme in the word. The fitted model contained one coefficient for each synthetic face phone me and one coefficient for each real face phoneme, and was a good fit to the data (*r*^2^ = 0.85, *p* < 10^−16^). In the hypothetical best case, the improved synthetic versions of */th/*, */dh/*, */f/*, */v/* would be as good as the real face versions. This was simulated in the model by replacing the synthetic face coefficients for these phonemes with the real face coefficients. With this adjustment, the model predicted a word accuracy of 43%, compared with 29% for the original versions of */th/*, */dh/*, */f/*, */v/*, suggesting that improving the quality of the synthetic faces for the four phonemes with very low synthetic benefit could significantly boost overall accuracy.

### Effects of alternative stimulus material

The overall phoneme accuracy rates shown in Figure 2C were determined by the phonemic content of the 64 tested words. This raises the question of how the results might differ for a much larger corpus of words. With a large enough corpus, the frequency of phonemes should match their overall prevalence in the English language. To simulate an experiment with a large corpus, we weighted the real-synthetic difference for each phoneme by its prevalence in the English language (Hayden, 1950). This procedure predicted an overall auditory-only phoneme accuracy of 40%, compared with 37% for the actual stimulus set. The predicted synthetic face phoneme accuracy was 60%, compared with 58% for the actual stimulus set, and the predicted real face accuracy was 78%, compared with 79% for the tested words. The predicted real-synthetic difference was 18% compared with 21% for the actual stimulus set. Testing with a large corpus of words (or a smaller set of words that matched overall English-language phoneme frequency) should lead to only small changes in accuracy.

### Differences between synthetic face types

**Figure 4:**
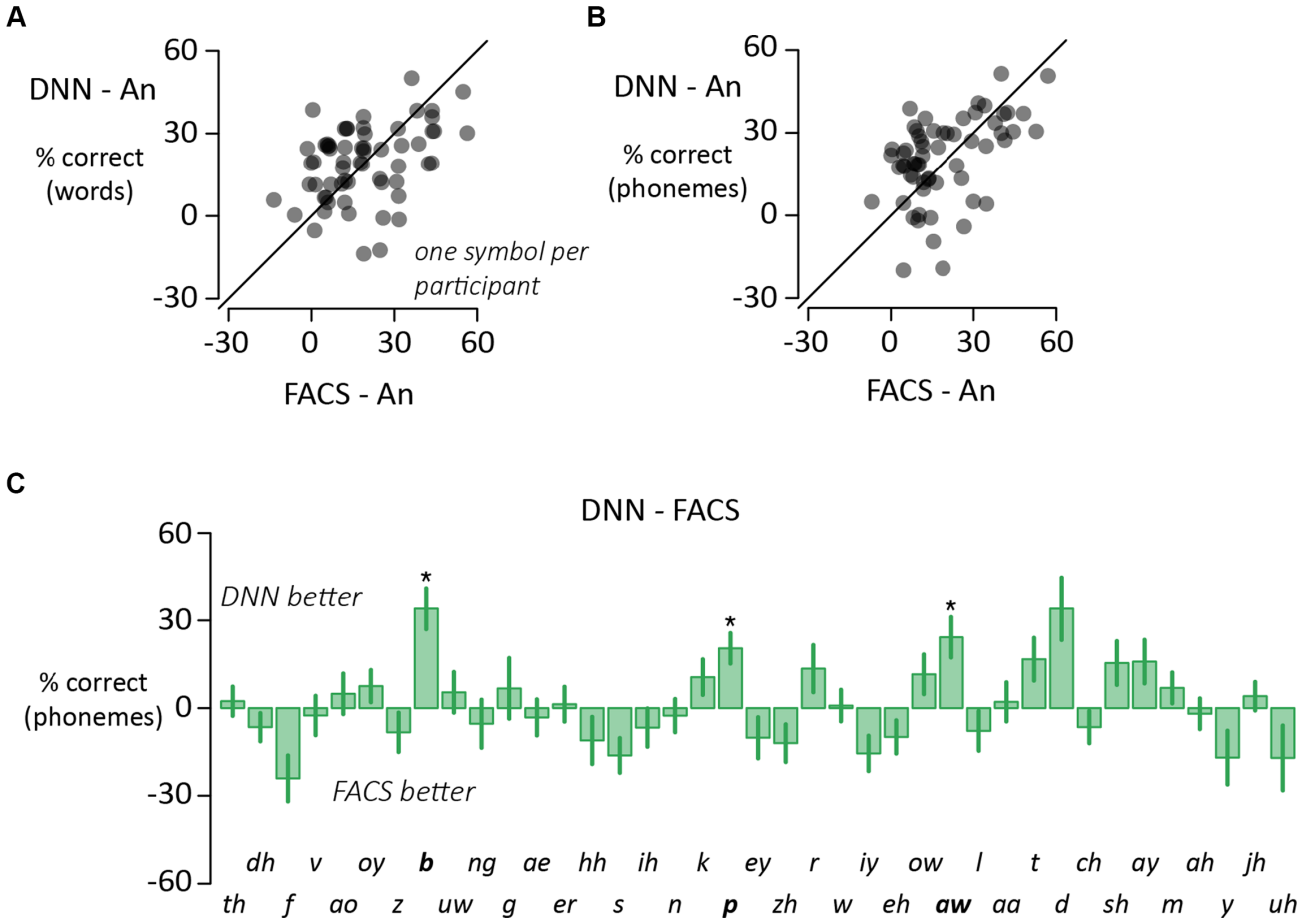
**(A)** Variability across participants for synthetic faces assessed with word-level scoring (one symbol per participant). The *y*-axis shows the perceptual benefit of synthetic DNN faces (DNN minus auditory-only accuracy). The *x*-axis shows the perceptual benefit of synthetic FACS faces (FACS minus auditory-only). **(B)** Variability across participants for synthetic faces assessed with phoneme-level scoring (one symbol per participant). For phoneme-level scoring, the phonemic content of the stimulus and response were compared and the percentage of correct phonemes calculated. **(C)** For each phoneme, the perceptual accuracy was calculated separately for DNN and FACS synthetic faces and subtracted (positive values indicate higher accuracy for DNN than FACS, negative values the opposite). Three phonemes (boldface labels, and star) showed significant difference in accuracy between formats (*p* < 0.05, corrected for multiple comparisons). Phoneme order identical to Figure 3A to facilitate comparison.

The accuracy for DNN and FACS faces was similar for the word analysis (Figure 4A; *t* = 0.2, *p* = 0.99) and the phoneme analysis (Figure 4B; *t* = 0.9, *p* = 0.82) leading us to combine both conditions into a single “synthetic face” accuracy for the analyses presented above. To search for more subtle differences between the synthetic face types, we compared DNN and FACS accuracy for individual phonemes (Figure 4C). 51% (20 out of 39) of the phonemes had higher accuracy for DNN faces vs. FACS faces, but after correction for multiple comparisons, the difference was significant for only three phonemes (*/b/*, */p/* and */aw/*), all with higher accuracy for DNN faces. An examination of word videos containing these phonemes did not reveal any obvious differences between the mouth movements of FACS and DNN faces.

### Individual differences

There was substantial variability in the benefit of visual speech to noisy speech perception across individuals. To determine if these individual differences were consistent, we correlated the visual benefit for different face types. For word accuracy (Figure 2B), there was a strong correlation across participants between the benefit of real and synthetic faces (*r* = 0.49, *p* = 10^−4^). The same was true for phoneme accuracy (Figure 2C; *r* = 0.71, *p* = 10^−10^). Comparing the two types of synthetic faces, there was a positive correlation between the benefit of DNN and JALI faces for both word accuracy (Figure 4A; *r* = 0.39, *p* = 0.002) and phoneme accuracy (Figure 4B; *r* = 0.47, *p* = 10^−4^).

## Discussion

Our study replicates decades of research by showing that seeing the face of a real talker improves speech-in-noise perception (Sumby and Pollack, 1954; Peelle and Sommers, 2015). Our study also confirms two recent reports that viewing a synthetic face generated by a deep neural network (DNN) significantly improves speech-in-noise perception (Shan et al., 2022; Varano et al., 2022). Both the present study and these previous reports found that the improvement from viewing DNN faces was only about half that provided by viewing real faces.

To determine if some idiosyncrasy of DNN faces was responsible for their poor performance relative to real faces, we also tested synthetic faces generated with a completely different technique, the facial action coding system (FACS). Since FACS explicitly models the relationship between the facial musculature and visual speech movements, we anticipated that it might provide more benefit to speech perception than DNN faces. Instead, the perceptual benefit of FACS faces was very similar to that of DNN faces.

Across participants, there was a positive correlation between the benefit of real and synthetic faces, leading us to infer that observers extract similar visual speech information from both kinds of faces, but that less visual speech information is available in synthetic faces. To better understand the real-synthetic difference, we decomposed stimulus words and participant responses into their component phonemes. This analysis revealed variability across phonemes. Four phonemes (*/th/*, */dh/*, */f/*, */v/*) had an especially large real-synthetic difference, driven by low performance for both DNN and FACS faces. Examining single frames of the real and synthetic videos for words containing these phonemes revealed an obvious cause for the reduced benefit of synthetic faces. The synthetic videos were missing the interactions between teeth, lip and tongue that are the diagnostic visual feature for */th/*, */dh/*, */f/*, */v/*. Without these visual cues, participants did not receive the visual information beneficial for detecting these phonemes in speech-in-noise.

### The concept of visemes

Visemes can be defined as the set of mouth configurations used to pronounce the phonemes in a language and may be shared across different phonemes. For instance, (*/f/*, */v/*) are visually similar labiodental fricatives that require talkers to place the top teeth on the lower lip. The acoustic difference is generated by voicing (*/f/* is unvoiced while */v/* is voiced); this voicing difference does not provide a visible cue to an observer. Similarly, (*/th/*, */dh/*) are dental fricatives, articulated with the tongue against the upper teeth, with */th/* unvoiced and */dh/* voiced. While there is no generally agreed upon set of English visemes, five common viseme classifications all place (*/f/*, */v/*) in one viseme category and (*/th/*, */dh/*) in a different viseme category (Cappelletta and Harte, 2012). Our results confirm the veracity of this grouping.

### How to improve synthetic faces

For the FACS faces, it would be possible to manually control teeth and tongue positioning using the underlying 3D face models, although this would be a time-consuming process. Alternately, the automated software used to animate the 3D face models (JALI)(Edwards et al., 2016) could be modified to automatically code teeth and tongue positioning. For DNN faces, it is less straightforward to incorporate dental and labial interactions. The DNN models are trained on thousands or millions of examples of auditory and visual speech, and the network learns the correspondence between particular sounds and visual features. It may be that dental and labial interactions are highly variable across talkers, or not easily visible in the videos used for training, resulting in their absence in the final output. A common step in neural network model creation is fine-tuning. Incorporating training data that explicitly includes dental and labial features, such as from electromagnetic articulography (Schönle et al., 1987) or MRI (Baer et al., 1987), would improve the DNN’s ability to depict these features.

### Other factors

While four phonemes showed an especially large real-synthetic difference (mean of 48% for */th/*, */dh/*, */f/*, */v/*) there was also a substantial difference for the remaining phonemes (mean of 16%). The origin of this difference is likely to be multi-faceted. One likely contributing factor is that just as for */th/*, */dh/*, */f/*, */v/*, the diagnostic mouth features for other phonemes are not as accurately depicted or as obvious in the synthetic faces as they are in the real face videos. This possibility could be tested by showing the most diagnostic single frame from each type of video and asking participants to guess the phoneme being spoken. The prediction is that, even for single video frames, synthetic performance would be worse than real face performance. Differences between DNN faces and real faces when pronouncing particular phonemes has been proposed as a method to detect deep-fake videos (Agarwal et al., 2020) although the phonemes examined in their study (*/m/*, */b/*, */p/*) were not those that showed the largest real-synthetic difference in the present study.

For real talkers, information about speech content is available throughout the face. Since synthetic face generation concentrates on the mouth and lip region, this decreases the information about speech content available to observers (Munhall and Vatikiotis-Bateson, 2004).

Another contributing factor for the real-synthetic difference could be temporal asynchrony, since the temporal alignment between auditory and visual speech contributes to causal inference and other perceptual processing underlying speech perception (Magnotti et al., 2013; Bhat et al., 2015). Synthetic faces were aligned to the auditory speech recording using a software processing pipeline; the JALI software used to create the FACS faces claims an audiovisual alignment accuracy of 15 ms (Edwards et al., 2016). Asynchrony produced by dubbing synthetic faces and real voices could be replicated for the real face condition by dubbing real faces and auditory recordings from a different talker or a synthesized voice.

### Relevance to experimental studies of audiovisual speech perception

An important reason for creating synthetic talking faces is to investigate the perceptual and neural properties of audiovisual speech perception (Thézé et al., 2020a,b). In the well-known illusion known as the McGurk effect, incongruent auditory and visual speech leads to unexpected percepts (McGurk and MacDonald, 1976). However, different McGurk stimuli vary widely in their efficacy. For instance, Basu Mallick et al. (2015) tested 12 different McGurk stimuli used in published studies, and found that the strongest evoked the illusion on 58% of trials while the weakest stimulus evoked the illusion on only 17% of trials. The causes of high inter-stimulus variability are difficult to study, as talkers are limited in their ability to control the visual aspects of speech production. In contrast, synthetic faces, especially those created with FACS and related techniques, provide the ability to experimentally manipulate visual speech (Thézé et al., 2020a; Shan et al., 2022; Varano et al., 2022) making them a key tool for improving our understanding of the McGurk effect other incongruent audiovisual speech (Dias et al., 2016; Shahin, 2019).

### Individual differences

Two findings of the present study are consistent with decades of research. First, that seeing the face of the talker is beneficial for noisy speech perception and second, that the degree of benefit varies widely between individuals (Sumby and Pollack, 1954; Erber, 1975; Grant et al., 1998; Tye-Murray et al., 2008; Van Engen et al., 2014, 2017; Peelle and Sommers, 2015; Sommers et al., 2020). Individual differences are observed even when participants’ eye movements are monitored, ruling out the trivial explanation that participants with low visual benefit fail to look at the visual display (Rennig et al., 2020). However, eye movements to particular parts of the talker’s face (specifically, a preference for foveating the mouth of the talker when viewing clear speech), combined with recognition performance during auditory-only noisy speech, explain about 10% of the variability across individuals (Rennig et al., 2020). At the neural level, fMRI response patterns in superior temporal cortex to clear and noisy speech are more similar in participants with a larger benefit from seeing the face of the talker (Rennig and Beauchamp, 2022; Zhang et al., 2023).

### Limitations of the present study

The present study has a number of limitations. In order to maximize the number of tested words and minimize experimental time, only a single noise level was tested, as in a previous study of DNN faces (Varano et al., 2022). A high level of noise (−12 dB) was selected to maximize the benefit of visual speech (Ross et al., 2007; Rennig et al., 2020). Another previous study of DNN faces tested multiple noise levels and found a lawful relationship between different noise levels and perception (Shan et al., 2022). As the amount of added auditory noise decreased, accuracy increased for the no-face, real face and DNN face conditions in parallel, converging at ceiling accuracy for all three conditions when no auditory noise was added. We would expect a similar pattern if our experiments were repeated with different levels of auditory noise. Another experimental approach is to present visual-only speech without any auditory input, although this condition differs from most real-world situations with the exception of profound deafness. The ability to extract information about speech from the face of the talker (known as lipreading or speechreading) varies widely across individuals, and it may be possible to improve this ability through training (Auer and Bernstein, 2007; Bernstein et al., 2022).

While our study only examined speech perception, a similar approach could be taken to compare real and synthetic faces in other domains, such as emotions and looking behavior (Miller et al., 2023).

## Conclusion

Massaro and Cohen (1995) pioneered the use of synthetic faces to examine audiovisual speech perception and recent advances in computer graphics and deep neural faces show that synthetic faces offer a promising tool for both research and practical applications to help patients with deficits in speech perception.

## Data availability statement

The original contributions presented in the study are included in the article/Supplementary material, further inquiries can be directed to the corresponding author.

## Ethics statement

The studies involving humans were approved by Institutional Review Board of the University of Pennsylvania, Philadelphia, PA. The studies were conducted in accordance with the local legislation and institutional requirements. The ethics committee/institutional review board waived the requirement of written informed consent for participation from the participants or the participants’ legal guardians/next of kin because Participants were recruited and tested online. Written informed consent was obtained from the individual(s) for the publication of any potentially identifiable images or data included in this article.

## Author contributions

YY: Writing – original draft, Writing – review & editing. AL: Writing – original draft, Writing – review & editing. YZ: Writing – original draft, Writing – review & editing. JM: Writing – original draft, Writing – review & editing. MB: Writing – original draft, Writing – review & editing.

## Footnotes

^1^https://www.mturk.com/

^2^http://www.speech.cs.cmu.edu/cgi-bin/cmudict

^3^https://jaliresearch.com/

^4^https://www.d-id.com/
