# Supplementary material for: Synthetic faces generated with the facial action coding system or deep neural networks improve speech-in-noise perception, but not as much as real faces
Source: Front Neurosci. 2024 May 9;18:1379988. doi: 10.3389/fnins.2024.1379988 (PMC11111898; doi:10.3389/fnins.2024.1379988)
Supplement: Supplementary file 2 [file Data_Sheet_1.zip › full_results_real_vs_syn copy.html]

The Effect on Speech-in-Noise Perception of Real Faces and Synthetic Faces Generated with either Deep Neural Networks or the Facial Action Coding System


Code 

- Show All Code
- Hide All Code

# The Effect on Speech-in-Noise Perception of Real Faces and Synthetic Faces Generated with either Deep Neural Networks or the Facial Action Coding System

#### Yingjia Yu, Anastasia Lado, Yue Zhang, John F. Magnotti, Michael S. Beauchamp

- 1 Code setup
  - 1.1 Required libraries and markdown
    setup
  - 1.2 Helper functions
- 2 Load data, get descriptive
  statistics
  - 2.1 Check accuracy on target
    trials
  - 2.2 Aggregate non-target data across
    subjects
- 3 Condition-level accuracy
  - 3.1 Show aggregate data by
    condition
- 4 Compare AV improvement by movie
  type
  - 4.1 Calculate improvement
  - 4.2 Plot for improvement
- 5 LME to compare conditions
  - 5.1 Word-level (binary) accuracy per
    trial
  - 5.2 Phoneme-level (continuous)
    accuracy per trial
- 6 Accuracy by phoneme
  - 6.1 Calculate identification accuracy
    for each phoneme
    - 6.1.1 Compare real vs. syn on
      individual phoneme detection rates
  - 6.2 Plot relative av improvement by
    phoneme
    - 6.2.1 Print out example words for top
      phonemes
  - 6.3 Predict word-level accuracy using
    phoneme-level predictors
    - 6.3.1 Estimate potential synthetic
      boost
    - 6.3.2 Real vs. Synthetic with
      real-world phoneme frequencies
    - 6.3.3 Deatiled comparison between DNN
      and FACS

# 1 Code setup

Mixed-effects models were fit using the R package `lme4.`
ANOVA-like tables (Analysis of Deviance tables using type II Wald 2
tests) were calculated using the `car` package (function
`Anova`). `emmeans` was used for post-hoc
contrasts.

`magrittr` is used for syntactic ease.
`future`, `future.apply`, `memoise` are
used to speed up processing. `readxl` is used to load XLSX
files. `stringr` is used for string processing.

## 1.1 Required libraries and markdown setup

```
stopifnot(all(
  sapply(c('lme4', 'car', 'emmeans', 'readxl', 'magrittr', 'stringr', 'knitr', 'future', 'future.apply', 'memoise'), 
         require, quietly = TRUE, character.only=TRUE)
))

# these functions are only used to produce the markdown file, but are
# included here for completeness
knitr::opts_chunk$set(eval = TRUE, fig.align = 'center', class.source='fold-hide')

# override the head function for pretty printing
head <- function(x, ...) {
  knitr::kable(utils::head(x, ...), format.args=list(digits=2))
}

# override the Anova function for pretty printing
Anova <- function(mod, ..., caption) {
  # fix up results a bit to avoid printing p=0
  res <- car::Anova(mod, ...)
  
  if(missing(caption)) {
    
    caption = 'car::Anova Analysis of Deviance table'
    if('glmerMod' %in% class(mod)) {
      
      f <- mod@call$formula
      f <- paste0(as.character(f)[c(2,1,3)], collapse=' ')
      
      caption = paste(caption, 'for', f)
    }
  }
  
  pv = pchisq(res$Chisq, df = res$Df, lower.tail = FALSE)
  res$`Pr(>Chisq)` = format.pval(pv, digits = 2, eps = 1e-32)
  
  knitr::kable(res, format.args=list(digits=1), caption=caption)
}

# kable doesn't like matrix output from aggregate
fix_agg_columns <- function(m) {
  to_fix <- which(sapply(m, is.matrix))
  for(ii in seq_along(to_fix)) {
    
    mat = m[[to_fix[ii]]]
    for(v in colnames(mat)) {
      m[[paste0(names(to_fix)[ii], '_', v)]] = mat[,v]
    }
  }
  m[to_fix] = NULL
  return(m)
}

str_to_arr <- function(w) {
  stringr::str_split(w, ' ')
}
```

## 1.2 Helper functions

```
remove_numerals <- function(ph) {
  gsub(pattern='[0-9]', ph, replacement = '')
}

do_aggregate <- function(x, ...) {
  aggregate(..., data=x)
}

# replace duplicated phonemes with a number indicating how many times 
# it has occurred in the word. 
#
# > fix_duplicates(c("EH", "EH" ,"AH" ,"AY", "EH"))
# [1] "EH"  "EH2" "AH"  "AY"  "EH3"
#

fix_duplicates <- function(set) {
  new_set <- set
  i <- 2
  while(any(dupes <- duplicated(new_set))) {
    dupes <- duplicated(new_set)
    new_set[which(dupes)] <- paste0(set[which(dupes)], i)
    i <- i + 1
  }
  
  return(new_set)
}

calculate_jaccard_similarity <- function(set1, set2) {
  
  set1 <- fix_duplicates(set1)
  set2 <- fix_duplicates(set2)
  
  length(intersect(set1, set2)) / length(union(set1, set2))
}


get_word_level_accuracy <- function(w, r) {
  as.integer(tolower(w) == tolower(r))
}

get_phoneme_level_accuracy <- function(word_ph, response_ph) {
  #shortcut!
  if(word_ph==response_ph) return(1)
  
  
  word_ph <- str_split(remove_numerals(word_ph),' ')[[1]]
  
  response_ph <- str_split(remove_numerals(response_ph),' ')[[1]]
  
  calculate_jaccard_similarity(word_ph, response_ph)
}

rbind_list <- function (ll) do.call(rbind, ll)

as_pdf <- function (fname, w, h, expr, to_pdf = F, bg = "white")  {
  if (isTRUE(to_pdf)) {
    on.exit(dev.off())
    fname <- fix_pdf_name(fname)
    pdf(fname, width = w, height = h, useDingbats = FALSE, 
        bg = bg)
  }
  
  invisible(eval(expr))
}


fix_pdf_name <- function (fname) {
  if (!grepl("\\.pdf$", fname)) {
    fname = paste0(fname, ".pdf")
  }
  return(fname)
}


ruta_axis <- function (side, at, tcl = -0.3, labels = at, las = 1, cex.axis = 1.3, 
                       cex.lab = 1.3, mgpy = c(3, 0.6, 0),
                       mgpx = c(3, 0.75, 0), ...)  {
  if (length(side) > 1) {
    return(invisible(sapply(side, ruta_axis, at = at, tcl = tcl, 
                            labels = labels, cex.axis = cex.axis, las = las, 
                            cex.lab = cex.lab, ...)))
  }
  mgp <- mgpy
  if (side%%2) 
    mgp <- mgpx
  invisible(as.matrix(axis(side, at = at, labels = labels, 
                           tcl = tcl, mgp = mgp, cex.axis = cex.axis, las = las, 
                           cex.lab = cex.lab, ...)))
}


plot_clean <- function (xlim, ylim=xlim, x = 1, y = 1,type = "n", xlab = "", ylab = "", 
                        ...) 
{
  plot(x, y, type = type, axes = F, ylab = ylab, xlab = xlab, 
       xlim = range(xlim), ylim = range(ylim), ...)
}


ebars.y <- function (x, y, sem, length = 0.05, up = T, down = T, code = 2, ...) {
  if (up) {
    arrows(x0 = x,
           y0 = as.numeric(y), y1 = as.numeric(y + sem),
           angle = 90, code = code, length = length, ...)
  }
  if (down) {
    arrows(x0 = x,
           y0 = as.numeric(y), y1 = y - sem,
           angle = 90, code = code, length = length, ...)
  }
}

ebars <- function (x, y = NULL, sem = NULL, length = 0.05, type = "n", 
                   col = "black", pt.col = col, code = 0, lwd=2, pt.lwd=1, ...) {
  if (is.null(y)) {
    if (is.matrix(x)) {
      y <- x[, 1]
      sem <- x[, 2]
    }
    else {
      y <- x
    }
    x <- seq_along(y)
  }
  if (is.matrix(y)) {
    sem <- y[, 2]
    y <- y[, 1]
  }
  if (is.null(sem)) {
    sem <- y
    y <- x
    x <- seq_along(y)
  }
  ind = (sem>0) & is.finite(sem)
  
  ebars.y(x[ind], y[ind], sem[ind], length, code = code, col = col, lwd=lwd, ...)
  
  points(x, y, type = type, col = pt.col, lwd=pt.lwd, ...)
}

lighten <- function(col, amt=0.5) {
  if(length(col) > 1) return(sapply(col, lighten, amt=amt))
  
  # cc = c(col2rgb(adjustcolor(col, amt), alpha = TRUE))
  
  cc <- c(col2rgb(col))
  
  new_color <- 255 * (
    cc/255 * (amt) + c(1,1,1)*(1-amt)
  )
  
  rgb(new_color[1], new_color[2], new_color[3], maxColorValue = 255)
}

do_staggered_labels <- function(xp, lbls, min.line=-1, max.line=0, cex=0.75) {
  n=seq_along(xp)-1
  
  lbls %<>% tolower
  
  odd = 2*n+1; odd = odd[odd <= length(lbls)]
  evn = odd+1; evn = evn[evn <= length(lbls)]
  
  axis(1, lwd=0, at=xp[odd], labels = lbls[odd], line=min.line, cex.axis=cex, font=3)
  axis(1, lwd=0, at=xp[evn], labels = lbls[evn], line=max.line, cex.axis=cex, font=3)
}

#### calculations
m_se <- function (x,scl=100, digits=4) {
  
  if (length(x) == 1) 
    return(scl*c(mean = x, se = 0))
  
  round(scl*c('mean' = mean(x, na.rm=TRUE), 'se' = se(x)), digits = digits)
}

not_NA <- function(x) !is.na(x)

se <- function (x, na.rm = TRUE)  {
  n <- sum(not_NA(x))
  re <- if (n < 2) {
    0
  }
  else {
    stats::sd(x, na.rm = na.rm)/sqrt(n)
  }
  if (is.na(re)) {
    re <- 0
  }
  return(re)
}
```

# 2 Load data, get descriptive statistics

```
all_data <- readxl::read_excel('full_data_Apr_2_2024.xlsx', sheet='data')

# calculate word-level accuracy
wla <- mapply(memoise(get_word_level_accuracy),
              all_data$word, all_data$response)

# calculate phoneme level accuracy
pla <- mapply(memoise(get_phoneme_level_accuracy),
              all_data$word_cmu_phonemes, all_data$response_cmu_phonemes)

## identify homophony
ind <- which(pla==1 & wla < 1)
wla[ind] = 1

all_data$word_level_accuracy = wla
all_data$phoneme_level_accuracy = pla

# get final sample size
cat('Total number of unique subejcts:', length(unique(all_data$subject)))
```

```
## Total number of unique subejcts: 61
```

```
# subjects per batch
kable(caption='Subjects per batch', aggregate(
  subject ~ batch, function(x) length(unique(x)), data=all_data
))
```

Subjects per batch

| batch | subject |
| --- | --- |
| exp1\_b1 | 15 |
| exp1\_b2 | 16 |
| exp1\_b3 | 15 |
| exp1\_b4 | 15 |

```
# create a palette that we can use
colors <- (c('gray70', 'orange3', 'orange1', 'dodgerblue3', 'orange2'))
names(colors) = c("real An", "jali AnV", "did AnV", "real AnV", 'syn AnV')
```

## 2.1 Check accuracy on target trials

```
target_trial_performace <- aggregate(word_level_accuracy ~ subject,
                                     sum,
                                     subset=all_data$condition=='target',
                                     data=all_data)

n.target_trials <- aggregate(word_level_accuracy ~ subject,
                             length,
                             subset=all_data$condition=='target',
                             data=all_data) %$% unique(word_level_accuracy)

# assert same number (9) of trials for everyone
stopifnot(length(n.target_trials) == 1)

perf <- round(d=3, 100*mean(target_trial_performace$word_level_accuracy)/n.target_trials)
cat("Mean performance on target trials: ", perf, '\n')
```

```
## Mean performance on target trials:  98.179
```

```
tbl <- table(target_trial_performace$word_level_accuracy)
xp <- barplot(tbl,
              las=1, border='black', col='gray40', axes=F,
              main='Performance on target trials\n(out of 9)',
              ylab='Frequency', xlab='# Correct'
)
ruta_axis(2, at=0:2*25)
text(xp, tbl+2, tbl, font=2, xpd=T)
```

## 2.2 Aggregate non-target data across subjects

```
measures <- c('word_level_accuracy', 'phoneme_level_accuracy')

all_measures_by_subject <- sapply(measures, function(m) {
  res <- aggregate(as.formula(sprintf(
    "%s ~ movie_type + subject + condition", m
  )),
  FUN=mean,
  subset=all_data$condition !='target',
  data=all_data)
  
  res$movie_type %<>% factor(levels=c('FACS', 'DNN', 'Real'))
  return(res)
}, simplify = FALSE)

agg_data_by_measure <- sapply(measures, function(nm) {
  aggregate(
    as.formula(sprintf("cbind(%s) ~ movie_type + condition", nm)),
    m_se,
    data=all_measures_by_subject[[nm]]
  )
}, simplify = FALSE)

kable(fix_agg_columns(agg_data_by_measure$word_level_accuracy),
      caption = 'Word-level accuracy by condition. SEM is over subjects')
```

Word-level accuracy by condition. SEM is over
subjects


| movie\_type | condition | word\_level\_accuracy\_mean | word\_level\_accuracy\_se |
| --- | --- | --- | --- |
| Real | An | 10.2459 | 1.1414 |
| FACS | AnV | 29.0984 | 1.8300 |
| DNN | AnV | 29.5082 | 1.5402 |
| Real | AnV | 58.9139 | 2.4771 |

```
kable(fix_agg_columns(agg_data_by_measure$phoneme_level_accuracy),
      caption = 'Phoneme-level accuracy by condition. SEM is over subjects')
```

Phoneme-level accuracy by condition. SEM is over
subjects


| movie\_type | condition | phoneme\_level\_accuracy\_mean | phoneme\_level\_accuracy\_se |
| --- | --- | --- | --- |
| Real | An | 28.0676 | 1.4369 |
| FACS | AnV | 47.3026 | 1.6416 |
| DNN | AnV | 48.7103 | 1.3839 |
| Real | AnV | 72.4667 | 1.9544 |

# 3 Condition-level accuracy

## 3.1 Show aggregate data by condition

```
make_lineup <- function(mat, mar=c(2,2, 1, 1)) {
  nm <- tail(names(mat),1)
  mat$y = mat[[nm]]
  df <- aggregate(y ~ movie_type + condition, m_se,data=mat)
  par(mar=mar)
  xp <- barplot(df$y[,1], space=.25,
                ylab=str_replace_all(nm, '_', ' '),
                ylim=c(0,100), axes=F,
                main=str_replace_all(nm, '_', ' '),
                col=sapply(1:4, lighten), border=1:4, 
                names.arg = paste(df$condition, df$movie_type))
  
  ebars(xp, df$y[,1],
        df$y[,2],
        col = 1:4, code=0, lwd=2
  )
  
  # abline(h=0, col='black', xpd=F)
  axis(1, at=par('usr')[1:2], tcl=0, labels = FALSE)
  ruta_axis(2, at=0:2*50, labels = (0:2/2)*100)
}

palette(colors)
par(mfrow=c(1,2))
# make plots for the different accuracy measures (word/phoneme/viseme)
sapply(names(all_measures_by_subject), function(nm) {
  as_pdf(paste0('../figure_pieces/figure_2_', nm),
         w = 1.75, h = 1.75, {
           make_lineup(all_measures_by_subject[[nm]])
         })
}) %>% invisible
```

# 4 Compare AV improvement by movie type

## 4.1 Calculate improvement

```
# Compare AV Improvement at the single subject level
calculate_av_improvement <- function(sbj, nm) {
  an = sbj[[nm]][sbj$condition=='An']
  
  realAV = sbj[[nm]][sbj$condition=='AnV' & sbj$movie_type=='Real']
  jaliAV = sbj[[nm]][sbj$condition=='AnV' & sbj$movie_type=='FACS']
  didAV = sbj[[nm]][sbj$condition=='AnV' & sbj$movie_type=='DNN']
  
  c('AVimp_Real' = realAV - an,
    'AVimp_FACS' = jaliAV - an,
    'AVimp_DNN' = didAV - an)
}

# note that we're calculating AV improvement for 
# word-level and phoneme-level accuracy in one go
av_improvement_by_type <- mapply(function(x, nm) {
  sapply(split(x, x$subject), calculate_av_improvement, nm=nm) %>% t
}, all_measures_by_subject, names(all_measures_by_subject),
SIMPLIFY = FALSE)

# average AV improvement
sapply(av_improvement_by_type, function(x) round(100*colMeans(x), 1))
```

```
##            word_level_accuracy phoneme_level_accuracy
## AVimp_Real                48.7                   44.4
## AVimp_FACS                18.9                   19.2
## AVimp_DNN                 19.3                   20.6
```

```
# create a single synthetic improvement score
av_improvement <- lapply(av_improvement_by_type, function(x) {
  syn_av <- rowMeans(x[,2:3])
  cbind('AVimp Real' = x[,1], 'AVimp Synth'=syn_av)
}) 

# handy print function
count_bin <- function(bool){
  paste(sum(bool, na.rm = TRUE), "out of", sum(!is.na(bool)))
}

cat("Whole-Word accuracy: how many show AV > A?\n\t", count_bin(av_improvement$word_level_accuracy[,'AVimp Real'] > 0)
)
```

```
## Whole-Word accuracy: how many show AV > A?
##   60 out of 61
```

```
cat("how many did better with real vs. synthetic?\n\t",
    count_bin(av_improvement$word_level_accuracy[,'AVimp Real'] > av_improvement$word_level_accuracy[,'AVimp Synth']))
```

```
## how many did better with real vs. synthetic?
##   59 out of 61
```

```
### for phoneme-level accuracy
cat("Phoneme-level accuracy: how many show AV > A?\n\t", count_bin(av_improvement$phoneme_level_accuracy[,'AVimp Real'] > 0))
```

```
## Phoneme-level accuracy: how many show AV > A?
##   61 out of 61
```

```
cat("how many did better with real vs. synthetic?\n\t",
    count_bin(av_improvement$phoneme_level_accuracy[,'AVimp Real'] > av_improvement$phoneme_level_accuracy[,'AVimp Synth']))
```

```
## how many did better with real vs. synthetic?
##   61 out of 61
```

```
### subject level correlations
cor.test(av_improvement$word_level_accuracy[,1], av_improvement$word_level_accuracy[,2])
```

```
## 
##  Pearson's product-moment correlation
## 
## data:  av_improvement$word_level_accuracy[, 1] and av_improvement$word_level_accuracy[, 2]
## t = 4.2624, df = 59, p-value = 7.391e-05
## alternative hypothesis: true correlation is not equal to 0
## 95 percent confidence interval:
##  0.2658879 0.6567889
## sample estimates:
##       cor 
## 0.4852202
```

```
cor.test(av_improvement$phoneme_level_accuracy[,1], av_improvement$phoneme_level_accuracy[,2])
```

```
## 
##  Pearson's product-moment correlation
## 
## data:  av_improvement$phoneme_level_accuracy[, 1] and av_improvement$phoneme_level_accuracy[, 2]
## t = 7.7096, df = 59, p-value = 1.697e-10
## alternative hypothesis: true correlation is not equal to 0
## 95 percent confidence interval:
##  0.5557295 0.8148664
## sample estimates:
##      cor 
## 0.708412
```

## 4.2 Plot for improvement

```
plot_comparison <- function(x,y, ..., mar=c(2,2.5,1,1), lim=range(pretty(c(x,y))), ticks) {
  
  par(pch=16, pty='s', las=1, mar=mar)
  plot_clean(lim, ...)  
  ruta_axis(1:2, ticks, cex.axis = .75)
  points(x, y, col=adjustcolor('black', alpha.f = .5))
  abline(0,1)
  abline(h=0, lty=2)
}

par(mfrow=c(1,2))
# set seed for jitter
set.seed(0311)
sapply(names(av_improvement), function(nm) {
  as_pdf(paste0('../figure_pieces/real_vs_syn_', nm),
         w=2, 2, {
           avimp <- av_improvement[[nm]]
           plot_comparison(x=100*jitter(avimp[,2], amount = .005),
                           main=str_replace_all(nm, '_',' '),
                           xlab = 'Synthetic AV Imp',
                           ylab = 'Real AV Imp',
                           y=100*jitter(avimp[,1], amount = .005),
                           lim=-10:90,
                           ticks=c(-10, 0, 45, 90)
           )
           if(nm=='word_level_accuracy') {
             title(ylab='Accuracy', line=1.5)
           }
         })
}) %>% invisible
```

# 5 LME to compare conditions

## 5.1 Word-level (binary) accuracy per trial

```
#ensure factors
all_data$movie_type %<>% factor
all_data$condition %<>% factor

# remove target condition
for_lme_wo_target <-subset(all_data, condition != 'target')

for_lme_wo_target$lme_condition <- paste0(for_lme_wo_target$movie_type, for_lme_wo_target$condition)
for_lme_wo_target$lme_condition <- 
  factor(for_lme_wo_target$lme_condition) %>% relevel(ref='RealAn')

res <- lmer(word_level_accuracy ~ lme_condition + (1|batch:subject) + (1|word),
            data = for_lme_wo_target)

## overall ANOVA table
Anova(res)
```

car::Anova Analysis of Deviance table

|  | Chisq | Df | Pr(>Chisq) |
| --- | --- | --- | --- |
| lme\_condition | 768 | 3 | <1e-32 |

```
emmeans::emm_options(lmerTest.limit = 5e3)
emmeans::emm_options(lmer.df = 'Satterthwaite')
post_hocs <- emmeans::emmeans(res, 
                              pairwise ~ lme_condition)

kable(post_hocs$contrasts, caption = 'Post hoc condition comparisons (Word-level accuracy)')
```

Post hoc condition comparisons (Word-level accuracy)


| contrast | estimate | SE | df | t.ratio | p.value |
| --- | --- | --- | --- | --- | --- |
| RealAn - DNNAnV | -0.1924854 | 0.0177286 | 3777.158 | -10.8573565 | 0.0000000 |
| RealAn - FACSAnV | -0.1895271 | 0.0177286 | 3777.158 | -10.6904866 | 0.0000000 |
| RealAn - RealAnV | -0.4856748 | 0.0177286 | 3777.158 | -27.3950300 | 0.0000000 |
| DNNAnV - FACSAnV | 0.0029584 | 0.0177286 | 3777.158 | 0.1668698 | 0.9983481 |
| DNNAnV - RealAnV | -0.2931894 | 0.0177286 | 3777.158 | -16.5376735 | 0.0000000 |
| FACSAnV - RealAnV | -0.2961477 | 0.0177286 | 3777.158 | -16.7045434 | 0.0000000 |

## 5.2 Phoneme-level (continuous) accuracy per trial

```
resPh <- lmer(phoneme_level_accuracy ~ lme_condition + (1|batch:subject) + (1|word),
              data = for_lme_wo_target)

## overall ANOVA table
Anova(resPh)
```

car::Anova Analysis of Deviance table

|  | Chisq | Df | Pr(>Chisq) |
| --- | --- | --- | --- |
| lme\_condition | 986 | 3 | <1e-32 |

```
kable(emmeans::emmeans(resPh, pairwise ~ lme_condition)$contrasts,
      caption = 'Post hoc condition comparisons (Phoneme-level accuracy)')
```

Post hoc condition comparisons (Phoneme-level
accuracy)


| contrast | estimate | SE | df | t.ratio | p.value |
| --- | --- | --- | --- | --- | --- |
| RealAn - DNNAnV | -0.2055750 | 0.0141407 | 3777.143 | -14.537865 | 0.0000000 |
| RealAn - FACSAnV | -0.1932883 | 0.0141407 | 3777.143 | -13.668969 | 0.0000000 |
| RealAn - RealAnV | -0.4427434 | 0.0141407 | 3777.143 | -31.309949 | 0.0000000 |
| DNNAnV - FACSAnV | 0.0122868 | 0.0141407 | 3777.143 | 0.868896 | 0.8209413 |
| DNNAnV - RealAnV | -0.2371684 | 0.0141407 | 3777.143 | -16.772084 | 0.0000000 |
| FACSAnV - RealAnV | -0.2494551 | 0.0141407 | 3777.143 | -17.640980 | 0.0000000 |

# 6 Accuracy by phoneme

## 6.1 Calculate identification accuracy for each phoneme

### 6.1.1 Compare real vs. syn on individual phoneme detection rates

```
agg_rs <- aggregate(cbind(correct, count) ~ phoneme + mtype + subject, FUN=sum, data=by_phoneme_accuracy, subset=mtype %in% c('Real', 'Syn'))

agg_rs$inc = agg_rs$count - agg_rs$correct

full_mod_rs <- glmer(family='binomial',
                     cbind(correct, inc) ~ phoneme*mtype + (1|subject),
                     data=agg_rs, control = glmerControl(optCtrl=list(maxfun=10000))
)
```

```
## Warning in (function (fn, par, lower = rep.int(-Inf, n), upper = rep.int(Inf, :
## failure to converge in 10000 evaluations
```

```
## Warning in optwrap(optimizer, devfun, start, rho$lower, control = control, :
## convergence code 4 from Nelder_Mead: failure to converge in 10000 evaluations
```

```
## Warning in checkConv(attr(opt, "derivs"), opt$par, ctrl = control$checkConv, :
## Model failed to converge with max|grad| = 0.0073251 (tol = 0.002, component 1)
```

```
car::Anova(full_mod_rs)
```

```
## Analysis of Deviance Table (Type II Wald chisquare tests)
## 
## Response: cbind(correct, inc)
##                Chisq Df Pr(>Chisq)    
## phoneme       937.01 38  < 2.2e-16 ***
## mtype         491.09  1  < 2.2e-16 ***
## phoneme:mtype 132.89 38  1.883e-12 ***
## ---
## Signif. codes:  0 '***' 0.001 '**' 0.01 '*' 0.05 '.' 0.1 ' ' 1
```

```
post_hoc <- emmeans::emmeans(
  full_mod_rs, pairwise ~ mtype, by='phoneme'
)

logodds_posthoc <- data.frame(summary(post_hoc$contrasts))

## how many show numeric advantage
cat("# (Real > Synth):", count_bin(logodds_posthoc$estimate < 0) )
```

```
## # (Real > Synth): 38 out of 39
```

```
## what about statistically significant after bonf correction?

# regrid to response scale to get the SE
# report all stats in the LOG ODDS
# this is only for the error bars on the graph
emm_contrasts <- emmeans(regrid(post_hoc), pairwise ~ mtype, by='phoneme')$contrasts

contrasts_for_barplot <- data.frame(summary(emm_contrasts))[,1:4]

# here were putting the p-values from the test in LO space into this object 
# so we have it handy
contrasts_for_barplot$p.value <- p.adjust(logodds_posthoc$p.value, method= 'bonf')

contrasts_for_barplot <- contrasts_for_barplot[order(contrasts_for_barplot$estimate, decreasing = FALSE),]

#flip sign for plotting
contrasts_for_barplot$estimate %<>% multiply_by(-1)
```

## 6.2 Plot relative av improvement by phoneme

```
# green_colors <- RColorBrewer::brewer.pal(3, 'Greens')[3:2]
green_colors <- c("#31A354", "#A1D99B")

.col = rep(green_colors, times=c(4,35))
as_pdf('../figure_pieces/accuracy_by_phoneme_rel_imp',  w=5.9, h=2.5, to_pdf=F,{
  par(mar=c(3,3,1,0))
  yy <- 100*contrasts_for_barplot$estimate
  sem = 100*contrasts_for_barplot$SE
  xp <- barplot(yy,
                border=.col, ylim=c(0,62), col=adjustcolor(.col, .5),
                axes=F, xlim = c(1.5,45.5),
                names.arg = NULL)
  ruta_axis(2, 0:2*30, cex.axis = 1)
  title(xlab='Phoneme', line=2, ylab='AV imp (Real - Synth)')
  do_staggered_labels(xp, cex = 0.75, contrasts_for_barplot$phoneme
  )
  axis(1, at=range(xp) + c(-1,1)*1, tcl=0, labels=F)
  
  #bonf.ps is already in sorted order
  ind <- which(contrasts_for_barplot$p.value<0.05)
  
  text(xp[ind], yy[ind]+sem[ind] + sign(yy[ind])*2, labels = '*', xpd=TRUE)
  
  ebars.y(xp, yy, sem, col = .col, code=0, lwd=2)
})
```

Now let’s consider just the top 4 phonemes vs. the rest

```
top4 = c('TH', 'DH', 'F', 'V')

agg_rs$phoneme_status <- ifelse(agg_rs$phoneme %in% top4, 'Top4', 'Other')

agg_top4 <- aggregate(cbind(correct,count) ~ phoneme_status + mtype + subject, FUN=sum, data=agg_rs)


agg_top4$accuracy = agg_top4$correct / agg_top4$count

kable(
  aggregate(accuracy ~ phoneme_status + mtype,
            m_se, data=agg_top4, d=0) %>% fix_agg_columns
)
```

| phoneme\_status | mtype | accuracy\_mean | accuracy\_se |
| --- | --- | --- | --- |
| Other | Syn | 61 | 1 |
| Top4 | Syn | 28 | 2 |
| Other | Real | 78 | 2 |
| Top4 | Real | 78 | 3 |

```
## real stim by phoneme type
t.test(accuracy ~ phoneme_status,
       paired=TRUE, data=agg_top4,
       subset=mtype == 'Real')
```

```
## 
##  Paired t-test
## 
## data:  accuracy by phoneme_status
## t = 0.24487, df = 60, p-value = 0.8074
## alternative hypothesis: true mean difference is not equal to 0
## 95 percent confidence interval:
##  -0.04561969  0.05834722
## sample estimates:
## mean difference 
##     0.006363767
```

```
## synth stim by phoneme type
t.test(accuracy ~ phoneme_status,
       paired=TRUE, data=agg_top4,
       subset=mtype == 'Syn')
```

```
## 
##  Paired t-test
## 
## data:  accuracy by phoneme_status
## t = 25.423, df = 60, p-value < 2.2e-16
## alternative hypothesis: true mean difference is not equal to 0
## 95 percent confidence interval:
##  0.3056868 0.3578984
## sample estimates:
## mean difference 
##       0.3317926
```

```
## real-syn diff for each phoneme type
bytype <- agg_top4 %>% split((.)$mtype, drop = TRUE)
stopifnot(all(bytype$Real[, c(1,3)] == bytype$Syn[, c(1,3)] ))

bytype$Diff = bytype$Real[,c(1,3)]
bytype$Diff$accuracy = bytype$Real$accuracy - bytype$Syn$accuracy

aggregate(accuracy ~ phoneme_status, mean, data=bytype$Diff)
```

```
##   phoneme_status  accuracy
## 1          Other 0.1706680
## 2           Top4 0.4960968
```

```
t.test(accuracy ~ phoneme_status, paired=T, data=bytype$Diff)
```

```
## 
##  Paired t-test
## 
## data:  accuracy by phoneme_status
## t = -11.317, df = 60, p-value < 2.2e-16
## alternative hypothesis: true mean difference is not equal to 0
## 95 percent confidence interval:
##  -0.3829489 -0.2679087
## sample estimates:
## mean difference 
##      -0.3254288
```

```
bp_with_e <- function(mse, main, .cols=RColorBrewer::brewer.pal(3, 'Greens')[3:2]) {
  xp <- barplot(mse$accuracy[2:1,1], axes=F, col=lighten(.cols), main=main,
                names.arg=c("top 4", 'other'),
                border=.cols, ylim=c(0,100), xlim=c(0,2.6))
  ebars(xp, mse$accuracy[2:1,1], mse$accuracy[2:1,2], col=.cols, code=0)
  ruta_axis(2, 0:2/.02)
  # abline(h=0, xpd=F)
  axis(1, at=par('usr')[1:2], tcl=0, labels = FALSE)
}

par(mfrow=c(1,3))
mapply(function(d, nm) {
  as_pdf(sprintf('../figure_pieces/barplot_inset_%s',nm), w=1.33, h=1.5, to_pdf = F, {
    par(mar=c(2,3.5,1,0.5))
    bp_with_e(d, nm)
    if(nm == 'Real-Synth') title(ylab='% Correct', line=2, cex.lab=1/0.667)
  })
}, lapply(bytype[c(3,2,1)], function(d) {
  aggregate(accuracy ~ phoneme_status, m_se, data=d, scl=100)
}), c('Real-Synth', 'Real', 'Synth'))
```

```
## $Diff
## NULL
## 
## $Real
## NULL
## 
## $Syn
## NULL
```

### 6.2.1 Print out example words for top phonemes

```
sapply(top4, function(fl) {
  all_data$word_cmu_phonemes %>% unlist %>%
    remove_numerals %>% str_detect(pattern=fl) %>%
    which ->ind
  
  all_data$word[ind] %>% unique
}) -> tbl

mapply(function(x, nm) {
  cat(nm, '\t:', paste0(collapse=', ', sort(x)), '\n')
}, tbl, names(tbl)
) %>% invisible
```

```
## TH   : booth, depth, ethic, moth, pathway, python, thank, theme, thoughts, with, youth 
## DH   : although, bathe, feathers, loathe, mouths, soothing, those, worthy 
## F    : chief, feathers, fetch, five, fusion, payoff 
## V    : five, heavy, invoice, version, voice, vouch, wives
```

```
# what if we remove these words from the database and check accuracy?
rem_words <- subset(all_data, !(word %in% unlist(tbl)))
kable(caption = 'Only words without top 4 phonemes', 
      aggregate(word_level_accuracy ~ movie_type + condition, mean, data=rem_words)
)
```

Only words without top 4 phonemes

| movie\_type | condition | word\_level\_accuracy |
| --- | --- | --- |
| Real | An | 0.1183971 |
| DNN | AnV | 0.3636364 |
| FACS | AnV | 0.3266423 |
| Real | AnV | 0.5355191 |
| DNN | target | 1.0000000 |
| FACS | target | 0.9781421 |
| Real | target | 1.0000000 |

```
# 
rem_words <- subset(all_data, (word %in% unlist(tbl)))
kable(caption = 'Only words with top 4 phonemes', 
      aggregate(word_level_accuracy ~ movie_type + condition, mean, data=rem_words)
)
```

Only words with top 4 phonemes

| movie\_type | condition | word\_level\_accuracy |
| --- | --- | --- |
| Real | An | 0.0819672 |
| DNN | AnV | 0.2065728 |
| FACS | AnV | 0.2453271 |
| Real | AnV | 0.6580796 |
| Real | target | 0.9508197 |

## 6.3 Predict word-level accuracy using phoneme-level predictors

```
# get a variable to allow us to combine DNN and FACS for this analysis
for_lme_wo_target$real_or_synth <- 
  factor(ifelse(for_lme_wo_target$lme_condition %in% c('RealAnV', 'RealAn'), 'Real', "Synth"))

word_mat <- aggregate(word_level_accuracy ~ word + real_or_synth + word_cmu_phonemes,
                      data=for_lme_wo_target,
                      function(x) c('Corr'=sum(x), 'Inc'=length(x)-sum(x)),
                      subset=lme_condition != 'RealAn')


word_mat$Correct = word_mat$word_level_accuracy[,'Corr']
word_mat$Inc = word_mat$word_level_accuracy[,'Inc']
word_mat$word_level_accuracy <- NULL

word_mat$av_acc <- with(word_mat, Correct / (Correct + Inc))

all_phonemes <- c("AA", "AE", "AH", "AO", "AW", "AY", "B", "CH", "D", "DH", "EH", 
                  "ER", "EY", "F", "G", "HH", "IH", "IY", "JH", "K", "L", "M", 
                  "N", "NG", "OW", "OY", "P", "R", "S", "SH", "T", "TH", "UH", 
                  "UW", "V", "W", "Y", "Z", "ZH")

build_ph_vec <- function(ph) {
  .X <- rep(0, length.out=length(all_phonemes))
  .X[
    which(all_phonemes %in% remove_numerals(str_split(ph, ' ')[[1]]))
  ] <- 1
  .X
}


Xmat <- t(sapply(word_mat$word_cmu_phonemes, build_ph_vec)) %>%
  set_colnames(all_phonemes)

pseudoR2 <- function(mod) {
  with(mod, 1-(deviance/null.deviance))
}

full_formula <- as.formula(paste0("cbind(Correct,Inc) ~ real_or_synth:(", paste0(all_phonemes, collapse='+'), ")"))

fitted.glm <- glm(full_formula,
                  family='binomial', data=cbind(word_mat, Xmat)
)
cat("Pseudo R2 for full model: ", round(digits=4, pseudoR2(fitted.glm)))
```

```
## Pseudo R2 for full model:  0.8455
```

```
# add predicted values into the data frame
word_mat$yhat_glm <- predict(fitted.glm, type='response')

# get a null model so we can calculate a p-value that corresponds
# to the above R2
null.glm <- glm(cbind(Correct,Inc) ~ 1,
                family='binomial', data=word_mat
)

anova(null.glm, fitted.glm, test = 'Chisq')
```

```
## Analysis of Deviance Table
## 
## Model 1: cbind(Correct, Inc) ~ 1
## Model 2: cbind(Correct, Inc) ~ real_or_synth:(AA + AE + AH + AO + AW + 
##     AY + B + CH + D + DH + EH + ER + EY + F + G + HH + IH + IY + 
##     JH + K + L + M + N + NG + OW + OY + P + R + S + SH + T + 
##     TH + UH + UW + V + W + Y + Z + ZH)
##   Resid. Df Resid. Dev Df Deviance  Pr(>Chi)    
## 1       127     987.52                          
## 2        49     152.57 78   834.95 < 2.2e-16 ***
## ---
## Signif. codes:  0 '***' 0.001 '**' 0.01 '*' 0.05 '.' 0.1 ' ' 1
```

### 6.3.1 Estimate potential synthetic boost

Estimate potential improvement if worst four phonemes from previous
step (\(/th/\), \(/dh/\), \(/f/\), and \(/v/\)) were boosted to “real” level
performance.

```
# find the betas to upgrade
modified_betas <- coef(fitted.glm)

to_modify <- c('TH', 'DH', 'F', 'V')

for(tt in to_modify) {
  ind <- which(str_detect(names(modified_betas), tt))
  real_ind <- which(str_detect(names(modified_betas[ind]), 'Real'))
  
  modified_betas[ind] <- modified_betas[ind[real_ind]]
}


# get the new predicted values
# Get the odds ratios first
X <- as.matrix(model.matrix(fitted.glm))
or <- exp(X %*% modified_betas)
word_mat$yhat_glm_mod <- c(or / (1+or))

aggregate(cbind('Yhat'=yhat_glm, 'YhatNew'=yhat_glm_mod, 'Orig'=av_acc) ~
            real_or_synth, 
          function(x)round(mean(x)*100,3),
          data=word_mat
)
```

```
##   real_or_synth   Yhat YhatNew  Orig
## 1          Real 59.087  59.087 58.88
## 2         Synth 29.271  42.957 29.36
```

### 6.3.2 Real vs. Synthetic with real-world phoneme frequencies

```
hayden <- readxl::read_excel('full_data_Apr_2_2024.xlsx', sheet='hayden1950')

# phoneme density estimates from Hayden 1950
hayden_phoneme_density <- hayden$PercentageOccurrence/100

# calculate frequencies of phonemes in our database
# get the phoneme frequencies in our dataset
phoneme_counts <- all_data$word_cmu_phonemes %>% 
  remove_numerals %>% str_split(' ') %>% unlist %>% table

local_phoneme_density <- c(phoneme_counts / sum(phoneme_counts))

by_phoneme_agg <- aggregate(accuracy ~ phoneme + mtype, mean, data=by_phoneme_accuracy) %>% split((.)$mtype)

sapply(by_phoneme_agg, function(bpa) {
  round(digits = 3, 100*c(
    'Hayden'=hayden_phoneme_density %*% bpa$accuracy,
    'YuEtAl'=local_phoneme_density %*% bpa$accuracy
  ))
}) -> result

kable(result,
      caption='Expected phoneme-by-phoneme accuracy based on phoneme frequency')
```

Expected phoneme-by-phoneme accuracy based on phoneme
frequency

|  | Ao | Syn | Real |
| --- | --- | --- | --- |
| Hayden | 40.351 | 60.336 | 78.088 |
| YuEtAl | 37.001 | 58.138 | 78.760 |

```
# Visualize the relative phoneme probabilities in the two datasets
# Note that our data is much more uniform
par(mfrow=2:1, mar=c(3,3,1,1))
do_bp <- function(h, ...) {
  xp <- barplot(h, ylim=c(0,0.1), axes=F, ylab='Prob(Phoneme)', ...)
  axis(2, 0:2/20, las=1)
  do_staggered_labels(xp, tolower(hayden$CMUPhoneme))
}

do_bp(hayden_phoneme_density, main='Hayden 1950')
do_bp(unname(local_phoneme_density), main='Local Data')
```

### 6.3.3 Deatiled comparison between DNN and FACS

```
lapply(names(av_improvement_by_type), function(nm) {
  print(nm)
  set.seed(04012024)
  as_pdf(paste0('../figure_pieces/FACS_vs_DNN_', nm),
         w=2, 2, to_pdf = F, {
           plot_comparison(lim = c(-30,60),
                           jitter(amount=100/64, 100*av_improvement_by_type[[nm]][,'AVimp_FACS']),
                           jitter(amount=100/64, 100*av_improvement_by_type[[nm]][,'AVimp_DNN']),
                           ticks = -1:2*30, xlab='FACS - An', ylab='DNN - An'
           )
           
           print(cor.test(
             av_improvement_by_type[[nm]][,'AVimp_FACS'],
             av_improvement_by_type[[nm]][,'AVimp_DNN']
           ))
         })
}) %>% invisible
```

```
## [1] "word_level_accuracy"
```

```
## 
##  Pearson's product-moment correlation
## 
## data:  av_improvement_by_type[[nm]][, "AVimp_FACS"] and av_improvement_by_type[[nm]][, "AVimp_DNN"]
## t = 3.2591, df = 59, p-value = 0.001857
## alternative hypothesis: true correlation is not equal to 0
## 95 percent confidence interval:
##  0.1539168 0.5848888
## sample estimates:
##       cor 
## 0.3905982 
## 
## [1] "phoneme_level_accuracy"
```

```
## 
##  Pearson's product-moment correlation
## 
## data:  av_improvement_by_type[[nm]][, "AVimp_FACS"] and av_improvement_by_type[[nm]][, "AVimp_DNN"]
## t = 4.1144, df = 59, p-value = 0.0001221
## alternative hypothesis: true correlation is not equal to 0
## 95 percent confidence interval:
##  0.2500931 0.6470596
## sample estimates:
##       cor 
## 0.4721768
```

```
# Get by-phoneme accuracy without combining DNN / FACS
by_phoneme_accuracy_by_type <- lapply(by_subj, accuracy_by_phoneme,
                                      combine_synth = FALSE) %>% rbind_list

agg_rs.synth <- aggregate(cbind(correct, count) ~ phoneme + mtype + subject, FUN=sum, data=by_phoneme_accuracy_by_type, subset=mtype %in% c('DNN', 'FACS'))

agg_rs.synth$inc = with(agg_rs.synth,
                        count - correct
)

full_mod_rs.synth <- glmer(family='binomial',
                     cbind(correct, inc) ~ phoneme*mtype + (1|subject),
                     data=agg_rs.synth, control = glmerControl(optCtrl=list(maxfun=10000))
)
```

```
## Warning in (function (fn, par, lower = rep.int(-Inf, n), upper = rep.int(Inf, :
## failure to converge in 10000 evaluations
```

```
## Warning in optwrap(optimizer, devfun, start, rho$lower, control = control, :
## convergence code 4 from Nelder_Mead: failure to converge in 10000 evaluations
```

```
## Warning in checkConv(attr(opt, "derivs"), opt$par, ctrl = control$checkConv, :
## Model failed to converge with max|grad| = 0.0107011 (tol = 0.002, component 1)
```

```
post_hoc.synth <- emmeans::emmeans(
  full_mod_rs.synth, pairwise ~ mtype, by='phoneme'
)

logodds_posthoc.synth <- data.frame(summary(post_hoc.synth$contrasts))

## how many show numeric advantage
cat("# (DNN > FACS):", count_bin(logodds_posthoc.synth$estimate > 0) )
```

```
## # (DNN > FACS): 20 out of 39
```

```
## what about statistically significant after bonf correction?

logodds_posthoc.synth$phoneme[
  which(p.adjust(logodds_posthoc.synth$p.value, method='bonf') < 0.05)
]
```

```
## [1] AW B  P 
## 39 Levels: AA AE AH AO AW AY B CH D DH EH ER EY F G HH IH IY JH K L M N ... ZH
```

```
# regrid to response scale to get the SE
# report all stats in the LOG ODDS
# this is only for the error bars on the graph
emm_contrasts.synth <- emmeans(regrid(post_hoc.synth), pairwise ~ mtype, by='phoneme')$contrasts

contrasts_for_barplot.synth <- data.frame(summary(emm_contrasts.synth))[,1:4]

contrasts_for_barplot.synth$p.value = p.adjust(logodds_posthoc.synth$p.value, method='bonf')

### order based on real vs. synth order
ph_ord <- contrasts_for_barplot$phoneme
rownames(contrasts_for_barplot.synth) = contrasts_for_barplot.synth$phoneme
contrasts_for_barplot.synth <- contrasts_for_barplot.synth[ph_ord,]

# green_colors <- RColorBrewer::brewer.pal(3, 'Greens')[3:2]
green_colors <- c("#31A354", "#A1D99B")

as_pdf('../figure_pieces/dnn_facs_by_phoneme_accuracy',  w=5.9, h=2.5, to_pdf=F,{
  par(mar=c(3,3,1,0))
  yy <- 100*contrasts_for_barplot.synth$estimate
  sem = 100*contrasts_for_barplot.synth$SE
  xp <- barplot(yy,
                border=green_colors[1], ylim=c(-60,60), col=adjustcolor(green_colors[1], .5),
                axes=F, xlim = c(1.5,45.5),
                names.arg = NULL)
  ruta_axis(2, -2:2*30, cex.axis = 1)
  title(xlab='Phoneme', line=2, ylab='AV imp (DNN - FACS)')
  do_staggered_labels(xp, cex = 0.75, contrasts_for_barplot.synth$phoneme, min.line = -2, max.line = -3
  )
  # axis(1, at=range(xp) + c(-1,1)*1, tcl=0, labels=F)
  
  #bonf.ps is already in sorted order
  ind <- which(contrasts_for_barplot.synth$p.value<0.05)
  
  text(xp[ind], yy[ind]+sem[ind] + sign(yy[ind])*4, labels = '*', xpd=TRUE)
  
  ebars.y(xp, yy, sem, col = green_colors[1], code=0, lwd=2)
})
```
